# Comparison between the Traditional and a Rapid Screening Test for Cryoimmunoglobulins Detection

**DOI:** 10.1155/2015/783063

**Published:** 2015-01-26

**Authors:** Federica Romitelli, Leopoldo Paolo Pucillo, Umberto Basile, Enrico Di Stasio

**Affiliations:** ^1^Institute of Biochemistry and Clinical Biochemistry, Catholic University of Sacred Heart, Largo Francesco Vito 1, 00168 Rome, Italy; ^2^I.N.M.I. “L. Spallanzani” I.R.C.C.S. Laboratorio di Biochimica Clinica e Farmacologia, Via Portuense 292, 00149 Rome, Italy; ^3^Department of Laboratory Medicine, Catholic University of Sacred Heart, Largo Francesco Vito 1, 00168 Rome, Italy

## Abstract

*Objectives*. A new rapid, automatic, and sensitive screening test useful to detect cryoglobulins in serum samples is proposed. *Design and Methods*. The increase of turbidity during the cryoglobulin aggregation was monitored spectrophotometrically in sera from 400 patients with clinical evidence of cryoglobulinemia related disorders and 100 controls. Results were correlated to those obtained by the traditional method. *Results*. Kinetics of the aggregation curves were described by their maximum turbidity increase, lag time, and slope. Despite a partial correspondence between the traditional and the rapid test, patients with symptomatic cryoglobulinemia showed turbidity values significantly higher than the determined cutoff. Moreover, a functional classification of cryoglobulins is proposed. *Conclusions*. Due to its high reproducibility, operator independence, low cost, and results obtained within 2 hours, the rapid test can be used as a “real time” monitoring of cryoglobulinemia related diseases and for the evaluation of plasmapheresis efficacy.

## 1. Introduction

Cryoglobulins are immunoglobulins that precipitate at temperature below 37°C and redissolve on rewarming [1–4]. The interest in these proteins has been growing because of their association with a number of diseases, including lymphoproliferative and autoimmune disorders and hepatitis C virus (HCV) infection [1, 5–10].

Immunochemical characterization of cryoglobulins has led to their classification into three major groups [11]. Type I represents individual monoclonal immunoglobulins, generally associated with lymphoproliferative diseases; type II consists of mixed immunoglobulins with a monoclonal component and is strongly associated with HCV infection; type III is constituted by a mixture of polyclonal immunoglobulins and is associated mainly with a wide range of infectious, autoimmune, and liver diseases.

Multiple but still poorly understood physiopathological mechanisms have been hypothesized for the comprehension of the cellular and molecular events leading to the clinical expression of cryoglobulinemic syndrome [9, 12–15]. In particular, it has been proposed that cryoprecipitation occurs because of the rapid formation of cold-insoluble IgM-IgG immune complexes [16, 17] or simply by a decreasing solubility phenomenon, resulting from an unfavorable interaction between cryoglobulins and solvent at low temperatures [18].

Stating that blood collection and transport procedures for cryoglobulin screening test remain a critical issue, also the methods used in clinical practice for the quantification of cryoglobulins are not standardized. Traditionally, cryoprecipitation is detected in vitro by incubating serum samples at low temperatures (4°C) for 2 to 15 days and the result of the assay is reported as a cryocrit, which is the percent of the precipitate in respect to the total serum volume [19]. However, different span time for sample inspection and poor sensitivity in appreciating low levels of cryoglobulins constitutes a severe limitation to the standardization and clinical interpretation of quantitative results in clinical practice [19–21].

In this paper we propose an alternative fast and easily reliable method for cryoglobulins detection in serum samples based on light scattering (turbidity). In respect to the traditional, time-consuming method, the performance of this rapid assay allows following the full kinetic process of cryoglobulin aggregation, before precipitation occurs. A comparative analysis between the traditional and the alternative screening test was performed on suspected cryoglobulinemic and noncryoglobulinemic sera, in order to evaluate the diagnostic sensitivity and specificity, as well as the advantages and the limitations of the two assays in clinical practice. Moreover, the kinetic analysis of the cryoaggregation process may provide a first attempt to a functional characterization of cryoglobulins, based on the comprehension of physiopathological mechanisms leading to cryoprecipitation.

## 2. Materials and Methods

### 2.1. Samples

Four hundred serum samples from patients with clinical evidence of different disorders related to cryoglobulinemia were studied by both methods. In particular, studies were conducted in parallel on sera, drawn for diagnostic purposes, from 100 patients affected by type I cryoglobulinemia related diseases (monoclonal gammopathies and Waldenstrom's macroglobulinemia), 250 subjects suspected of having cryoglobulinemia secondary to HCV infection, and 50 patients with autoimmune diseases (Sjogren's syndrome, rheumatoid arthritis). All patients were asymptomatic, with the exception of 15 subjects who had clinical signs of acute cryoglobulinemic syndrome. Moreover one hundred sera from healthy volunteers served as control group. The study was conducted according to the principles of the Helsinki declaration after approval by the local institution review board and written informed consent was obtained from all subjects.

### 2.2. Blood Sampling

Blood was drawn by venipuncture in Vacutainer tubes prewarmed at 37°C and left to clot for 2 hours at the same temperature; the serum was separated by centrifugation (2500 ×g for 10 min) at 37°C. An aliquot of each sample (1800 *μ*L) was immediately tested with the rapid screening assay and the remaining volume dispensed in a graduated Wintrobe tube for the traditional assay (time 0).

### 2.3. Cryoglobulins Determination by the Traditional Method

The presence of precipitate in the samples was determined by visual inspection, before centrifugation, after 1, 3, 7, and 15 days of cold incubation (4°C) and was expressed as percentage of the volume occupied by the precipitated proteins compared to the total volume of serum. Before the final quantification, a subjective scoring of cryoprecipitate was adopted: absence of cryoprecipitate was scored as “−” while cryoprecipitate <1%, ranging from 1 to 5% and >5%, was scored as “+,” “++,” and “+++,” respectively. On the 15th day of cold incubation, samples were centrifuged at 2500 ×g for 10 min at 4°C and the amount of cryoglobulins was estimated as “cryocrit %,” determined as the percentage of the volume occupied by the cryoprecipitate compared to the total volume of serum, after centrifugation occurred [19]. According to the cryocrit values obtained, samples were divided into three classes: samples with cryocrit values higher than 1.0%, with cryocrit values between 0.5 and 1.0%, and with absence of cryoglobulins.

### 2.4. Cryoglobulins Determination by the Rapid Screening Test

The rapid method for cryoglobulins determination is based on the detection of the scattered light by turbidimetry [22]. Measurements were performed using a Cary3 dual-beam spectrophotometer (Varian Australia Pty. Ltd., Mulgrave, Australia) on samples contained in a square cell (10 mm path length), whose temperature was maintained at 10°C. This temperature was chosen to prevent the misting of the cuvette walls in instruments without nitrogen flow and the temperature dependence of the aggregation curve is reported in a previous paper [22]. Briefly, an aliquot (1800 *μ*L) of serum samples, collected as described in Blood Sampling, was dispensed in a cuvette in a spectrophotometer, the system was blanked, and cryoglobulins aggregation was recorded reading the absorbance values at 350 nm, until precipitation occurred.

Three parameters were used to describe the evolution of the aggregation process over time: (1) *A*_max⁡_, the maximum absorbance value, directly related to the cryoglobulin concentration [22]; (2) the maximum slope (*δA*/*δt*) of the aggregation curve; (3) the lag time (*t*_0_) obtained from extrapolation of the maximum slope to the absorbance reading *A*_0_ [22].

According to the *A*_max⁡_ values, indicative of the quantitative measurement of cryoglobulins, samples were divided into three groups on the basis of percentile distribution in the control sera: the first group comprehended samples showing *A*_max⁡_ values ≤0.1 absorbance units (94% of control sera); the second group assembled samples with *A*_max⁡_ values ranging from 0.1 to 0.2 absorbance units (5% of control sera); finally, samples showing *A*_max⁡_ values >0.2 absorbance units were assigned to the third group (1% of control sera). On the basis of a rigorous application of a 95% confidence interval, 0.1 is the cutoff for positivity of the test.

In order to determine the diagnostic accuracy of rapid and traditional test for the presence of clinical symptoms of cryoglobulinemic syndrome we analyzed the ROC curves and calculated area under curves (AUCs). Cutoff values were determined at 90% sensitivity criterion derived directly from the ROC curves.

## 3. Results

### 3.1. Results of the Rapid and Traditional Test as a Function of the Visual Inspection of Cryocrit at Different Days of Incubation before Centrifugation

In Table 1, results obtained on cryoglobulinemic and controls (*n* = 500) samples by means of visual inspection at the 1st, 3rd, 7th, and 15th days of incubation and by the traditional (at the 15th day) and the rapid screening test (at time 0) are reported and compared. In addition, the number of samples for each cryoprecipitate score (see Section 2) is shown, together with the corresponding mean cryocrit value obtained after 15 days of cold incubation and the rapid test results at time 0.

At the first incubation day, in most samples (286, 57.2%) no cryoprecipitate could be observed by visual inspection. In forty (8%) cases it was possible to detect a cryoprecipitate (+ score), whose cryocrit was lower than 1% at the 15th day. In eighty-eight (17.6%) cases scored as ++ the cryocrit at the 15th day ranged from 1 to 5% and in 86 (17.2%) (score +++) it was higher than 5%. After fifteen days of cold incubation, the number of samples showing no cryoprecipitate decreased (252 versus 286) and, together, the number of samples with visible cryoprecipitate with a cryocrit positioning between 1 and 5% or higher than 5% increased (105 versus 88 and 101 versus 86 samples, resp.).

The performance of the rapid screening test at time 0 indicated that out of 252 samples negative for cryoglobulins with the traditional test, after 15 days of cold incubation, 230 showed absorbance values <0.1, 21 showed 0.1 < *A*_max⁡_ < 0.2, and one sample was frankly positive for cryoglobulins (*A*_max⁡_ > 0.2). Out of 42 samples with cryocrit lower than 1%, with the rapid test, 22 presented values of *A*_max⁡_ < 0.1 and 20 of 0.1 < *A*_max⁡_ < 0.2. Moreover, out of 105 samples with a cryocrit ranging from 1 to 5%, 11 showed *A*_max⁡_ < 0.1, 75 had absorbance values between 0.1 and 0.2%, and 19 were frankly positive (>0.2). Finally, all samples (101) showing by the traditional test cryocrit values >5% displayed Abs_max⁡_ levels higher than 0.2 with the rapid test.

### 3.2. Comparison of the Rapid and Traditional Test at the 15th Day of Incubation after Centrifugation

Table 2 shows the overall results obtained from the comparative analysis of the rapid and the traditional screening tests. According to the classification reported in Section 2, samples distribution for the traditional test showed that among the expected cryoglobulinemic samples 46% had cryocrit values >1%, 16% had cryocrit values between 0.5 and 1%, and no cryoprecipitate was detectable in 38% of samples and in all controls. Absorbance values ≥0.1, by means of the rapid test, were found in 94% (174 out of 185) of samples with frankly positive cryocrit, in 61% (41 out of 63) with low cryocrit (0.5–1%), and in 9% of those with absence of cryoprecipitate (16 patients plus 6 controls). The correlation between cryocrit and turbidimetric readings was found statistically significant (Pearson coefficient = 0.763, *P* < 0.001). However, despite this partial correspondence between the traditional and the rapid screening test, all of the patients with symptomatic cryoglobulinemic syndrome (*n* = 15) showed Abs_max⁡_ values higher than 0.5 (0.55–1.99; mean 1.17) and cryocrit ranging from 6.2 to 50 (mean 23).

**Figure 1:**
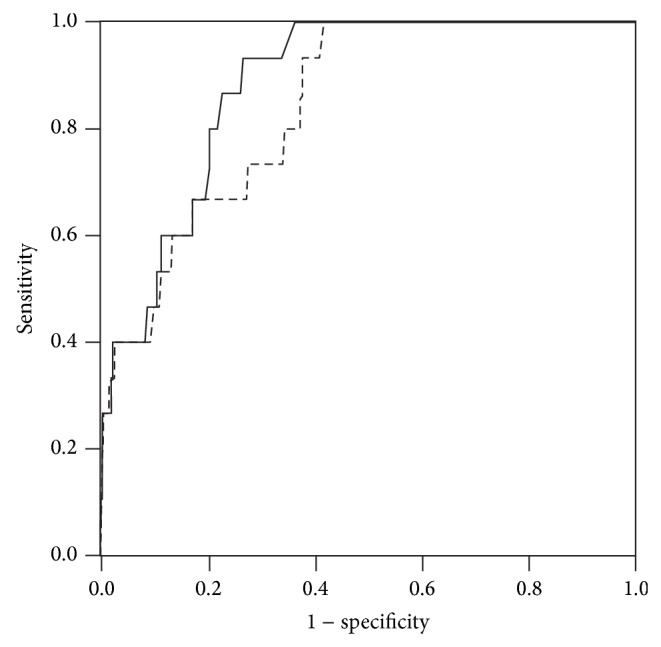
ROC curves of rapid (continuous line) and traditional (dotted line) tests in respect to the presence of clinical symptoms of cryoglobulinemic syndrome.

Sensitivity and specificity of the rapid screening test, calculated in respect to the results of the traditional one, were 87% and 91%, respectively. Moreover, diagnostic sensitivity and specificity of the rapid screening and the traditional test, limited in respect to the presence of clinical symptoms of cryoglobulinemic syndrome, were calculated by means of ROC curves shown in Figure 1. AUCs and cutoff values, determined at the 90% sensitivity, for rapid and traditional test were 0.88 ± 0.30 (*P* < 0.001) and 0.23 (specificity, 74.5%) and 0.84 ± 0.41 (*P* < 0.001) and 2.6 (specificity, 62.0%), respectively.

### 3.3. Cold-Induced Aggregation Curves of Type I and II Cryoglobulinemic Sera: Shape and Parameters

**Figure 2:**
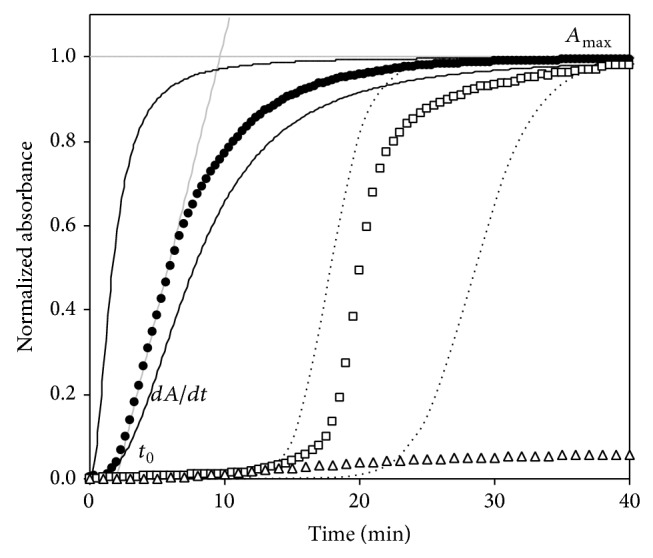
Typical aggregation curves obtained by the rapid screening test performed on type II (•) and type I (□) cryoglobulinemic sera and on a sample of a control subject (∆). Curves are normalized to the type II profiles; original *A*_max⁡_ values were 1.32, 1.40, and 0.08, respectively. When tested using the traditional method, the cryoglobulinemic sample showed a cryocrit value of about 5%. Continuous and dotted lines refer to samples of type II and I cryoglobulinemic sera displaying the lowest and highest recorded lag time (*t*_0_) values (*t*_0(min⁡⁡)_ = 0.2 and 2.9 and *t*_0_ = 15.4 and 24.1, resp.).

Figure 2 shows representative cold-induced aggregation curves obtained after performing the rapid screening test on type I and type II cryoglobulinemic serum samples and on a control serum sample normalized to the type II curve profile; moreover, parameters derived from the phenomenological analysis of the process are reported. These cryoglobulinemic samples shared a comparable cryocrit value (~5%) obtained with the traditional test. The comparison between the two plots showed that, despite a similar sigmoidal curve shape with comparable maximum slopes and asymptotic absorbance (*A*_max⁡_), the *t*_0_ parameter observed was profoundly different. In particular, type I cryoglobulinemic sample showed a lag time (*t*_0_) 10-fold higher than type II (*t*_0_ ratio = 10, where *t*_0_ ratio = (*t*_0_)_type I_/(*t*_0_)_type II_). Similar results were obtained comparing *t*_0_ values of all type I and type II cryoglobulins, achieving a mean *t*_0_ ratio of 12 ± 4. The aggregation curves of samples that have types II and III cryoglobulins did not show significantly different kinetic parameters (data not shown).

## 4. Discussion

The methods used to date for the quantification of cryoglobulins have not been uniform between different laboratories [19–21, 23]. Traditional tests are based on cryoprecipitate quantification after prolonged (2–21 days) cold incubation of serum samples. However, the performance of this assay in clinical laboratories poses considerable problems due to the lack of standardization of preanalytical and analytical procedures. Recently, Vermeersch et al. [19] evaluated the current practice in the detection, analysis, and reporting of cryoglobulins, by means of a questionnaire sent to 140 laboratories participating in the UK National External Quality Assessment Service quality control program. The study showed that only 36% of laboratories respect the standard preanalytical procedures to collect blood (tube preheating, transport in container, sedimentation, and/or centrifugation at 37°C) and a wide variation at many steps of the analytical procedure (timing for cryoprecipitation at 4°C, washing and/or resolubilization of cryoprecipitate, etc.) exists between different laboratories.

Another important problem encountered in performing the traditional test for cryoglobulin detection is due to the false-negative results. In fact, since the definition of cryoprecipitate presence depends on a visual inspection, a small amount of cryoglobulins in the sample could not be visible, and/or cryoglobulins with peculiar physical aspects, like a cryogel, could be missed.

An alternative test to detect cryoglobulins in serum samples was first proposed by Kalovidouris and Johnson [24] and recently reviewed by our group [22]. Kalovidouris and Johnson's assay was based on the spectrophotometric detection of a difference in optical density between two aliquots of serum sample incubated at 4°C and 37°C, respectively [24]. The cryoglobulin screening assay, defined here as “rapid screening test,” is based on the same physical principle (light scattering detection), although some modifications have been introduced. In our approach, a detailed phenomenological analysis of the entire aggregation process with the identification of parameters describing its evolution over time for each specimen was performed [22]. Moreover, the number of patients, as well as the one of control subjects, was notably higher than the number of cases reported by Kalovidouris and Johnson [24] and, in addition, sera from patients affected by different disorders related to cryoglobulin typing (25% type I, 62.5% type II, and 12.5% type III) were analyzed.

We compared data from the performance of both classical and rapid test for cryoglobulins screening on 500 serum samples. Only 1 (0.4%) and 21 (8.3%) out of 252 samples negative for cryoprecipitate showed Abs_max⁡_ ≥ 0.2 and between 0.1 and 0.2, respectively. The major discrepancies between the two screening tests were found within patients with cryocrit >1%. Out of 185 patients frankly positive with the traditional test, 6% were negative with the rapid test and 30% reported absorbance values slightly altered (0.1-0.2). It is noteworthy to highlight the different phenomenon monitored by the two assays: the traditional test visually detects the amount of precipitated aggregates, whereas the increase in the scattered light, on the basis of the rapid test, is due to the formation of aggregates, before precipitation occurs. Therefore, results of samples positive to the rapid test and negative to the classical one could find their explanation in (1) a higher sensitivity of spectrophotometer signal in respect to the macroscopic visual evaluation in detecting low amounts of aggregates/precipitate or (2) the possibility that cryoglobulins at low concentrations, when undergoing the aggregation process, do not reach an adequate cluster dimension for overcoming the solubility limit, thus not forming precipitate. On the contrary, the incongruence of samples positive to the traditional test and negative to the rapid one could be reasoned by the following: (1) the different span time of the two assays (i.e., days versus hours) which does not exclude the possibility of a slow rate of aggregation (that could be undetectable in the first hour of observation and culminate in the precipitate formation only after days of cold incubation) or (2) the occurrence of alternative aggregation mechanisms (such as a gel/network formation or presence of macroscopic not precipitated aggregates), which interfere with the spectroscopic readings. The last hypothesis is supported by the observation of a high and exclusive frequency (45%) of particular macroscopic aspects (gel consistency, homogeneous turbidity) in such samples.

A critical evaluation of such results has allowed us to propose a “functional” classification of cryoglobulins: (1) “fast” cryoglobulins: positive results obtained with the rapid test (showing an aggregation rate of minutes), as well as with the traditional test; “slow” cryoglobulins: only the traditional assay shows a positive result (precipitation rate of days); “early” cryoglobulins: only the rapid assay shows a positive result. The functional cryoglobulins classification reported above might be helpful in the clinical approach and management of patients. In fact, “fast” forms could be identified as proteins able to rapidly aggregate and candidate to precipitate also in vivo; “slow” forms could be identified as a laboratory finding of uncertain clinical relevance (probably artefacts) or as consequence of an unspecific precipitation originating from decrease of solubility of serum proteins after long cold incubation; “early” cryoglobulins could be identified as proteins found in samples at low concentrations, so that no visible precipitate could be detected with the classical method.

Finally, the utility of the rapid test might be considered in the therapeutic management of cryoglobulinemic patients whose medical treatment includes plasmapheresis. As known, plasmapheresis is a common procedure which transiently removes the circulating cryoglobulins reducing morbidities associated with such disorder [25, 26]. The efficacy of such treatment could be tested in a short time and in a large number of patients by means of the rapid test, enabling the patient to be treated by a different therapeutic approach if poorly effectual. The utility of this approach needs further investigations in studies supported by specific clinical protocols.

## 5. Conclusions

The present study proposes the rapid screening test as a reliable and sensitive method to use in a complementary way with the traditional test for the assessment of cryoglobulinemia. Despite many advantages in using the rapid test, in respect to the traditional one (high reproducibility, no examiner dependency, and low cost results obtained within 2 hours) and the possibility to discriminate monoclonal or mixed cryoglobulinemia by the *t*_0_ parameter, cryocrit and its immunological characterization by immunofixation remain fundamental for cryoglobulins typing, whereas the rapid test can be useful as a “real time” monitoring of the disease development and the evaluation of plasmapheresis efficacy.

## Figures and Tables

**Table 1:** Comparison of results obtained with the traditional and the rapid test. For the traditional assay, the number of samples (*n*) at different cryoprecipitate scores (see Section 2) by visual inspection at 1, 3, 7, and 15 days of cold incubation, the mean cryocrit %, after centrifugation, at the 15th day, and the corresponding *A*_max⁡_ value from the rapid screening test at time 0 are reported. The number of control samples is reported between brackets for the rapid and the traditional test at the 15th day of visual inspection.

Visual inspection	Traditional test					Rapid test (*A*_max⁡_)		
	Incubation days				Cryocrit % value at the 15th day	<0.1 (*n*)	0.1-0.2 (*n*)	≥0.2 (*n*)
	15 (*n*)	*7* (*n*)	*3* (*n*)	*1* (*n*)	mean ± SD [median; range]			
−	**252** (100)	*257 *	*261 *	*286 *	0 —	230 (94)	21 (5)	1 (1)
+	**42**	*40 *	*45 *	*40 *	0.74 ± 0.02 [0.75; 0.5–1]	22	20	—
++	**105**	*102 *	*93 *	*88 *	4.84 ± 0.30 [5.4; 0.5–9.9]	11	75	19
+++	**101**	*101 *	*101 *	*86 *	12.40 ± 1.20 [8.9; 0.5–56.5]	—	—	101

**Table 2:** Cross results from the performance of the traditional and the rapid cryoglobulin screening test. Data are reported as number of samples and percentage (between brackets).

	Cryoglobulinemic sera (*n* = 400)			Control sera (*n* = 100)
	Cryocrit > 1% (*n* = 185; 46%)	Cryocrit 0.5–1% (*n* = 63; 16%)	Absence of cryocrit (*n* = 152; 38%)	Absence of cryocrit (*n* = 100; 100%)
*A*_max⁡_	Patients (%)	Patients (%)	Patients (%)	Subjects (%)
<0.1	11 (6)	22 (34)	136 (89)	94 (94)
0.1-0.2	56 (30)	39 (61)	16 (11)	5 (5)
≥0.2	118 (64)	2 (3)	—	1 (1)
